# Squalene through Its Post-Squalene Metabolites Is a Modulator of Hepatic Transcriptome in Rabbits

**DOI:** 10.3390/ijms23084172

**Published:** 2022-04-10

**Authors:** Roubi Abuobeid, Javier Sánchez-Marco, María J. Felices, Carmen Arnal, Juan Carlos Burillo, Roberto Lasheras, Rebeca Busto, Miguel A. Lasunción, María Jesús Rodríguez-Yoldi, Roberto Martínez-Beamonte, Jesús Osada

**Affiliations:** 1 Departamento de Bioquímica y Biología Molecular y Celular, Facultad de Veterinaria, Instituto de Investigación Sanitaria de Aragón, Universidad de Zaragoza, E-50013 Zaragoza, Spain; roubi.a.obeid@gmail.com (R.A.); javiersanchezmarc@gmail.com (J.S.-M.); romartin@unizar.es (R.M.-B.); 2 Departamento de Farmacología, Fisiología, Medicina Legal y Forense, Facultad de Veterinaria, Instituto de Investigación Sanitaria de Aragón, Universidad de Zaragoza, E-50013 Zaragoza, Spain; mariajo_herrera@hotmail.com (M.J.F.); mjrodyol@unizar.es (M.J.R.-Y.); 3 Departamento de Patología Animal, Facultad de Veterinaria, Instituto de Investigación Sanitaria de Aragón, Universidad de Zaragoza, E-50013 Zaragoza, Spain; arnal@unizar.es; 4 Instituto Agroalimentario de Aragón, CITA-Universidad de Zaragoza, E-50013 Zaragoza, Spain; 5 CIBER de Fisiopatología de la Obesidad y Nutrición, Instituto de Salud Carlos III, E-28029 Madrid, Spain; rebeca.busto@hrc.es (R.B.); miguel.a.lasuncion@hrc.es (M.A.L.); 6 Laboratorio Agroambiental, Servicio de Seguridad Agroalimentaria de la Dirección General de Alimentación y Fomento Agroalimentario, Gobierno de Aragón, E-50071 Zaragoza, Spain; jcburillo@aragon.es (J.C.B.); rjlasheras@aragon.es (R.L.); 7 Servicio de Bioquímica-Investigación, Instituto Ramón y Cajal de Investigación Sanitaria (IRYCIS), Hospital Universitario Ramón y Cajal, E-28034 Madrid, Spain

**Keywords:** squalene, virgin olive oil, rabbits, murine, AML12 cell line, lipid droplets, transcriptome, liver, hepatic, RNA sequencing

## Abstract

Squalene is a natural bioactive triterpene and an important intermediate in the biosynthesis of sterols. To assess the effect of this compound on the hepatic transcriptome, RNA-sequencing was carried out in two groups of male New Zealand rabbits fed either a diet enriched with 1% sunflower oil or the same diet with 0.5% squalene for 4 weeks. Hepatic lipids, lipid droplet area, squalene, and sterols were also monitored. The Squalene administration downregulated 9 transcripts and upregulated 13 transcripts. The gene ontology of transcripts fitted into the following main categories: transporter of proteins and sterols, lipid metabolism, lipogenesis, anti-inflammatory and anti-cancer properties. When the results were confirmed by RT-qPCR, rabbits receiving squalene displayed significant hepatic expression changes of *LOC100344884* (*PNPLA3*), *GCK*, *TFCP2L1*, *ASCL1*, *ACSS2*, *OST4*, *FAM91A1*, *MYH6*, *LRRC39*, *LOC108176846*, *GLT1D1* and *TREH*. A squalene-enriched diet increased hepatic levels of squalene, lanosterol, dihydrolanosterol, lathosterol, zymostenol and desmosterol. Strong correlations were found among specific sterols and some squalene-changed transcripts. Incubation of the murine AML12 hepatic cell line in the presence of lanosterol, dihydrolanosterol, zymostenol and desmosterol reproduced the observed changes in the expressions of *Acss2*, *Fam91a1* and *Pnpla3*. In conclusion, these findings indicate that the squalene and post-squalene metabolites play important roles in hepatic transcriptional changes required to protect the liver against malfunction.

## 1. Introduction

**Figure 1:**
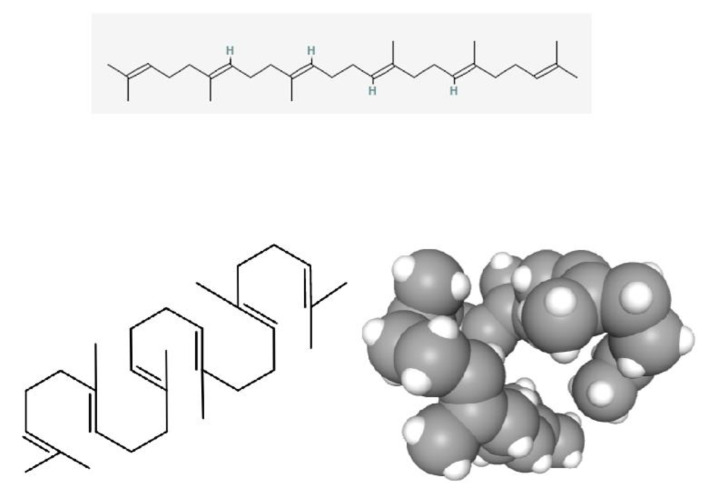
Squalene chemical structures. Obtained from https://pubchem.ncbi.nlm.nih.gov/compound/638072#section=2D-Structure, accessed on 9 February 2022 and https://www.lipidmaps.org/databases/lmsd/LMPR0106010002?LMID=LMPR0106010002, accessed on 9 February 2022.

The Seven Countries Study and subsequent epidemiological studies have linked the Mediterranean diet to natural occurring longevity, welfare and low rates of metabolic disorders [1,2]. There are some geographical modifications in the pattern of the Mediterranean diet; however, all of them use virgin olive oil (VOO) as a principal source of energy and as a health-promoting component [3]. Briefly, the consumption of VOO positively modulates the lipid metabolism, lipogenic response, insulin resistance, immune-inflammatory pathways, antithrombotic effect, blood pressure control, detoxification of reactive species and endothelial functions [3,4,5,6]. A substantial part of this effect has been attributed mainly to the minor unsaponifiable fraction [7,8], which represents about 0.5 to 1.5% of the oil and is composed of phytosterols, phenolic compounds, triterpenes and hydrocarbons. The latter accounts for almost 50% of the unsaponifiable composition [9,10], with squalene as the preeminent component [7]. This polyunsaturated terpenoid is made up of six units of isoprene (Figure 1) and is present in VOO at a concentration of 1.5 to 10.1 g per kg depending on cultivars, agronomical issues and olive fruit processing [11].

Several assays have tested favorable properties of squalene as a strong antioxidant, anti-inflammatory and highly effective oxygen scavenger against cell deterioration, senescence, neoplasm and chemotherapy-induced side-effects and as an enhancer of immune response to various associated antigens [11,12]. In humans, the estimated squalene intake ranges from 30 to 400 mg per day [13], with high oral absorption efficiency at rates from 60 to 85% compared to 42% in animals [14,15]. Dietary squalene is transported by chylomicrons into circulation, followed by hepatic uptake prior to conversion into sterols and bile acids or re-secreted into the bloodstream into very low-density lipoproteins (VLDL) and low-density lipoproteins (LDL) and distributed to various tissues [15].

*Oryctolagus cuniculus* represents an excellent laboratory model for its high absorption and responsiveness to dietary cholesterol [16] and its high cholesteryl ester transfer protein activity [17]. In addition, the liver secretes apolipoprotein APOB100 [18] and plasma APOB100-containing particles have similar chemical components to humans [11]. Several studies have tested the hepatic biological effects caused by dietary squalene. Kritchevsky et al. [19] were the first to prove that a diet enriched in squalene caused an increase in mass and unsaponifiable material in the liver. Recently, it has been observed that exogenous squalene fed to these animals results in an increase in the hepatic content of squalene in the rough endoplasmic reticulum, the nucleus and expanded lipid vesicle size [20,21]. These authors have also found a hepatic accumulation of non-esterified cholesterol and of sterol precursors in the modified Kandutsch–Russell pathway [21]. This initial complexity may point out a complex network of gene expressions being involved. To address this hypothetical setting, an RNA-sequencing approach was tackled in rabbits consuming squalene and murine cell lines treated with cholesterol precursors were used in an attempt to clarify sterol’s ability to modulate in vivo gene expression changes.

## 2. Results

### 2.1. Body Weight and Hepatic Parameters

**Figure 2:**
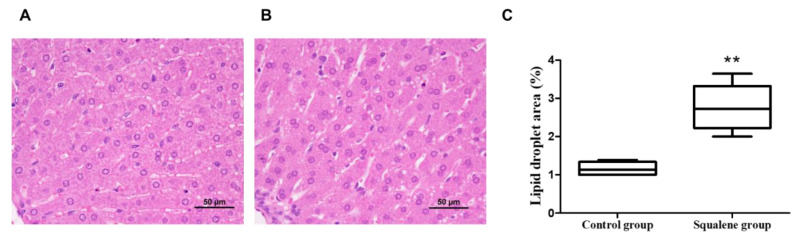
Hepatic histological analyses in rabbits fed with different diets. Representative liver micrographs when consuming control containing sunflower-oil diet (**A**) vs. 0.6 g/kg squalene-enriched control diet (**B**). Liver sections from each animal were stained with hematoxylin and eosin and evaluated blindly. Morphometric difference in amount of lipid droplet area in both control and squalene groups (**C**). Data are means and 10–90 percentiles for each group. Statistical analyses were performed according to Mann–Whitney’s U-test. **, *p* < 0.01.

**Figure 3:**
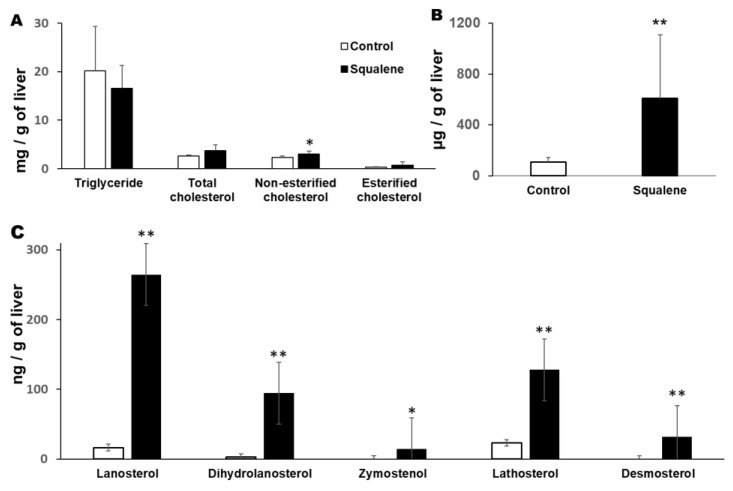
Hepatic lipid composition. (**A**) Triglyceride and cholesterol. (**B**) Squalene level. (**C**) Cholesterol biosynthesis intermediate sterols. Unfilled bar, control diet; filled bar, squalene-supplemented diet. Data are means ± SD. Statistical analyses were performed according to Mann–Whitney’s U-test. * *p* < 0.05, ** *p* < 0.01 vs. control.

Rabbits receiving the squalene-supplemented diet did not change body weight, despite a slight decline in food intake (Supplementary Figure S1). In addition, hepatic lipid droplet area was augmented (*p* < 0.01) (Figure 2), with an increase in the contents of non-esterified cholesterol content (*p* < 0.02), squalene (*p* < 0.01) and sterol metabolites such as lanosterol, dihydrolanosterol, zymostenol, lathosterol and desmosterol (for all *p* < 0.01), however, no significant change in triglycerides nor in esterified cholesterol was detected (Figure 3).

### 2.2. Hepatic Gene Expression

To determine the response of squalene intake to the hepatic transcriptome, RNA was extracted from five animals receiving the squalene-supplemented diet and from five receiving the control diet and sequenced by next-generation sequencing using the DNBseq platform. From each library, clean read sequences represented an average of 45.8 ± 26 × 10^6^ with a ratio of coverage of 91.60%. On average, 87.69% of the reads were mapped with the reference genome, and the uniformity of the mapping results for each sample suggests that the samples were uniform. In total, 19,163 genes were identified, of which 18,561 were known and 720 were new. Dietary squalene did not influence single nucleotide polymorphisms. Regarding transition, A-G were 55.6 ± 5.4 vs. 50.9 ± 5.9 and C-T were 55.7 ± 5.5 vs. 50.9 ± 5.9 for control and squalene groups, respectively. Regarding transversions, A-C were 9.2 ± 0.9 vs. 8.6 ± 0.7; A-T were 6.6 ± 0.8 vs. 6.4 ± 0.4; C-G were 10.8 ± 1.1 vs. 9.9 ± 1.0 and finally, G-T were 9.2 ± 1.0 vs. 8.6 ± 0.6 for control and squalene groups, respectively.

**Figure 4:**
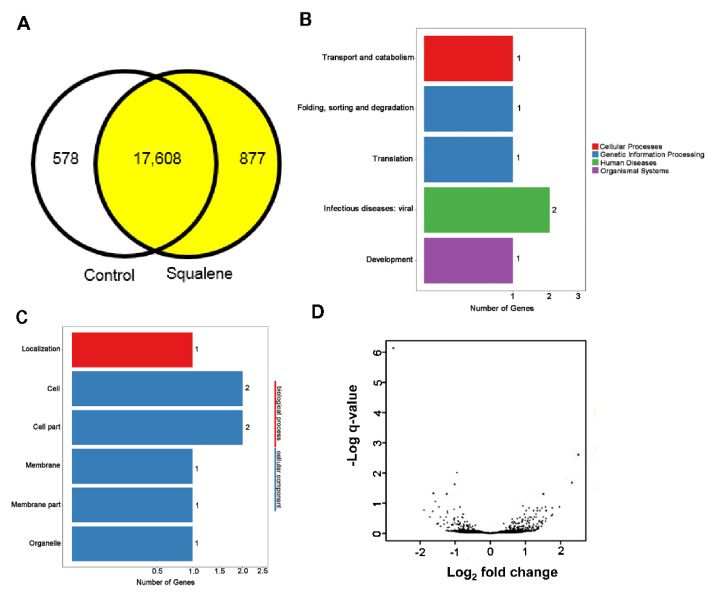
Differentially expressed genes (**A**) Venn diagram analysis. Control expressed 18,186 transcripts vs. 18,485 in squalene group. (**B**) Pathway classification of DEGs, *X* axis; number of DEG. *Y* axis; functional classification of KEGG. (**C**) GO classification of DEGs, axis represents number of DEG. *Y* axis represents GO term. (**D**) Volcano plot of DEGs. *X* axis, log_2_ transformed fold change. *Y* axis; −log_10_ of false discovery rate.

When alternative splicing events were tested, squalene administration had a significant influence on splicing events, alternative 5′ splicing, alternative 3′ splicing site and retained introns (data not shown), while no effect was found on skipped exons or mutually exclusive exons. Splicing patterns led to a variety of differentially splicing genes and a variety of different isoforms from one gene, with a total of 10,924 novel transcripts, of which 8562 were previously anonymous splicing events for known genes, 720 were novel coding transcripts without any known features, and the rest were 1642 long noncoding RNA. Differentially expressed genes were 18,485 in the squalene group and 18,186 in the control group as shown in Figure 4A. Gene ontology of upregulated and downregulated genes involves molecular, biological functions and cellular components. The latter required a larger number of genes, as revealed in Figure 4C. Summary of DEGs and volcano plot show DEGs distribution are displayed in Figure 4B,D. Using more stringent conditions (log_2_ fold change higher than 1.5 or less than −1.5) and a false discovery rate of *p* < 0.001, nine transcripts were downregulated (Table 1) and 13 transcripts were upregulated (Table 2). The biological function of some of these genes fitted into the following five main categories: hepatic transport of sterols and proteins, lipid metabolism, lipogenesis, anti-inflammatory and anti-cancer processes.

**Figure 5:**
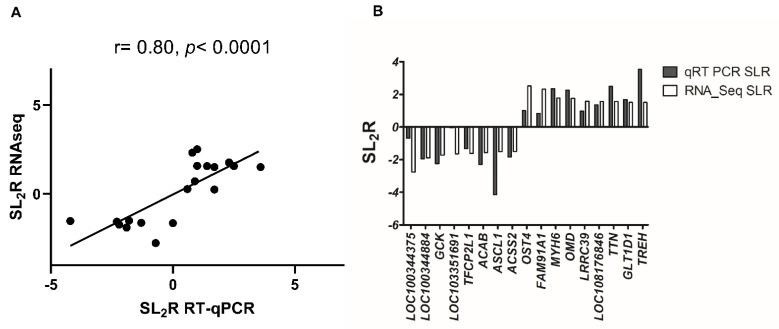
Concordance between used methods of RNA analysis. (**A**) Correlation analysis of 17 selected genes between RNAseq and RT-qPCR normalized to the invariant *PIPB* and *GAPDH* genes. The mean values obtained for signal log_2_ ratio (SL_2_R) from individual analyses (Table 3) were plotted against the RNAseq which used partially pooled samples (Table 1 and Table 2). Good agreement between the procedures was observed (r = 0.8, *p* < 0.0001). (**B**) Difference in results of SL_2_R expression of both procedures of the 17 selected genes.

To confirm the RNAseq data, 17 transcripts with log_2_ fold change higher than 1.5 or lower than −1.5 were randomly selected from Table 1 and Table 2, including *LOC100344375, LOC100344884* (*PNPLA3*), *GCK*, *LOC103351691*, *TFCP2L1*, *ACAB*, *ASCL1*, *ACSS2*, *OST4*, *FAM91A1*, *MYH6*, *OMD*, *LRRC39*, *LOC108176846*, *TTN*, *GLT1D1* and *TREH*. Their RT-qPCR assays were set up and verified on individual hepatic samples. A correlation study between RNAseq and RT-qPCR by assessing log_2_ fold change values of transcripts showed a significant agreement (r = 0.8, *p* < 0.0001) (Figure 5A) and all samples were properly classified (Figure 5B). In rabbits receiving squalene (Table 3), twelve transcripts displayed significant expression changes (*LOC100344884* (*PNPLA3*), *GCK*, *TFCP2L1*, *ASCL1*, *ACSS2*, *OST4*, *FAM91A1*, *MYH6*, *LRRC39*, *LOC108176846*, *GLT1D1* and *TREH*).

**Figure 6:**
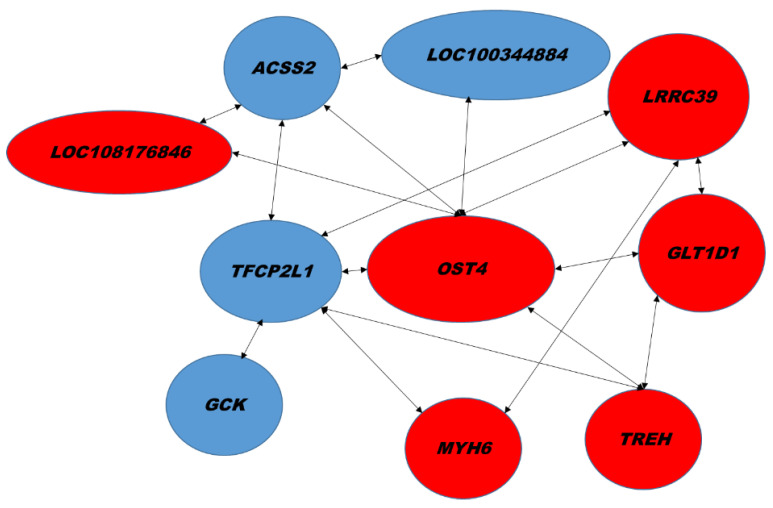
Network of hepatic transcripts. A, Association among transcript changes assayed by RT-qPCR. Red, up regulation. Blue, down regulation. Significant Spearman’s correlations (*p* < 0.02).

Network correlations obtained from RT-qPCR results revealed significant association among key transcripts (Figure 6). *OST4* and *TFCP2L* were hubs and correlated with *LOC100344884* (*PNPLA3*), *GCK*, *ACSS2*, *MYH6*, *LRRC39*, *LOC108176846*, *GLT1D1* and *TREH*.

**Figure 7:**
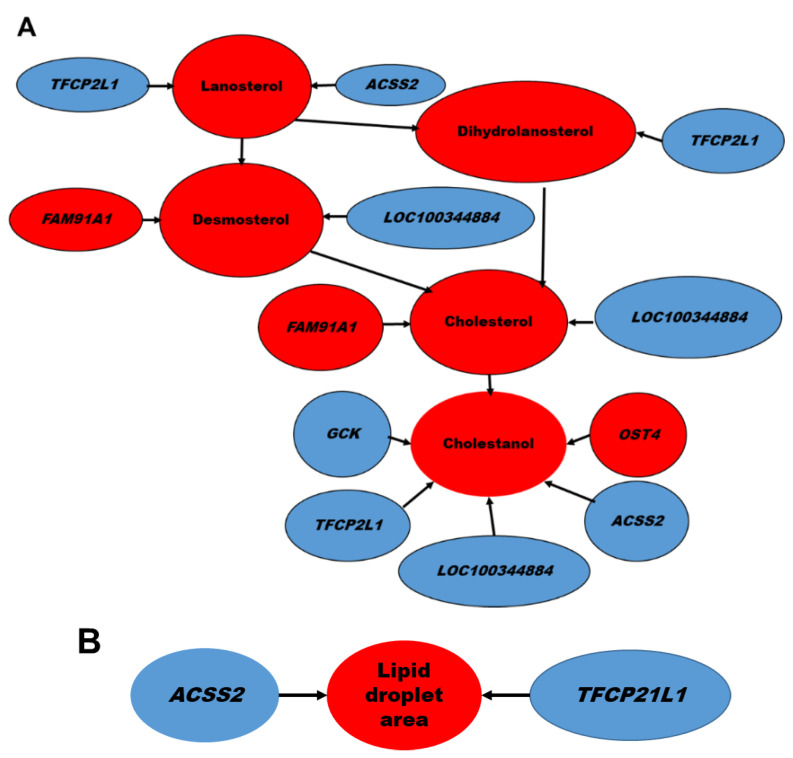
Correlation between hepatic transcripts and biological parameters in response to squalene intake. (**A**) transcripts associated with sterol precursors in the Kandutsch–Russell pathway. (**B**) transcripts related to lipid droplet area. Red; up regulation. Blue; down regulation. Significant (*p* < 0.02) Spearman’s correlations are shown.

Likewise, significant associations were observed among several transcripts and the hepatic content of metabolites in cholesterol biosynthetic pathways, including lanosterol, dihydrolanosterol, and desmosterol (Figure 7A). In this regard, down regulations of *TFCP2L1* and *ACSS2* were associated with sterols in the Kandutsch–Russell pathway and with increased hepatic lipid droplet area (Figure 7B). The increase in *FAM91A1* and the decrease in *LOC100344884* (*PNPLA3*) were associated with sterols in the Block pathway.

**Figure 8:**
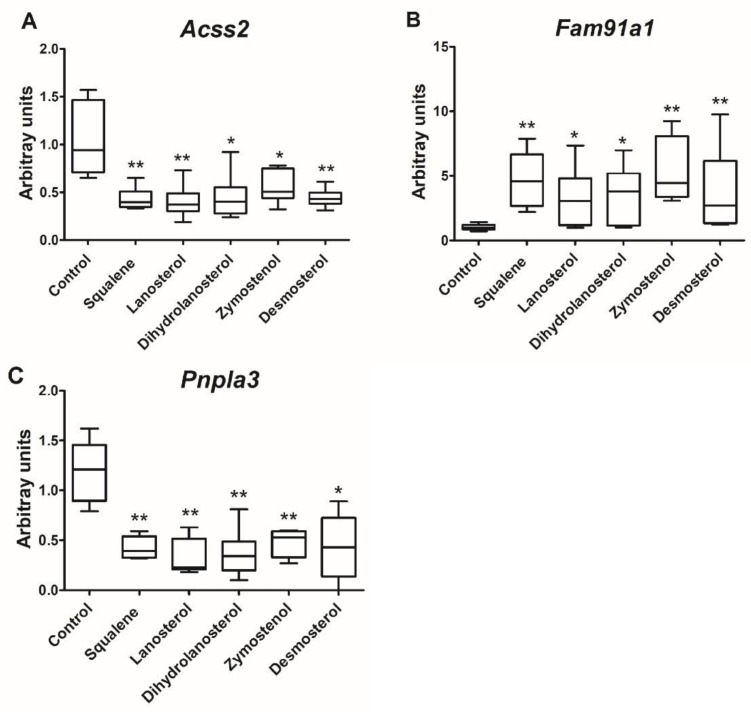
Effect of in vitro incubation of cholesterol precursors on selected gene expressions. AML12 cells were incubated in presence of 0.1% ethanol (control) or 200 µM squalene, 200 nM lanosterol, dihydrolanosterol, zymostenol or desmosterol dissolved in 0.1% ethanol for 6 h. The experiment was performed in triplicate, with *n* = 6 in each experiment for control and treated cells. Panels (**A**–**C**) correspond to *Acss2*, *Fam91a1* and *Pnpla3* mRNA levels normalized to *Pipb* by RT-qPCR, respectively. Data are means and 10–90% percentiles. Statistical analyses were performed according to Mann–Whitney’s U-test. * *p* < 0.05, ** *p* < 0.01 vs. control.

To verify those associations, a murine AML12 hepatic cell line was incubated with 200 µM squalene and 200 nM of lanosterol, dihydrolanosterol, zymostenol or desmosterol for 6 h. In the assayed conditions, only *Acss2*, *Fam91a1* and *Pnpla3* expressions reproduced the pattern observed in rabbit’s livers (Figure 8).

## 3. Discussion

**Figure 9:**
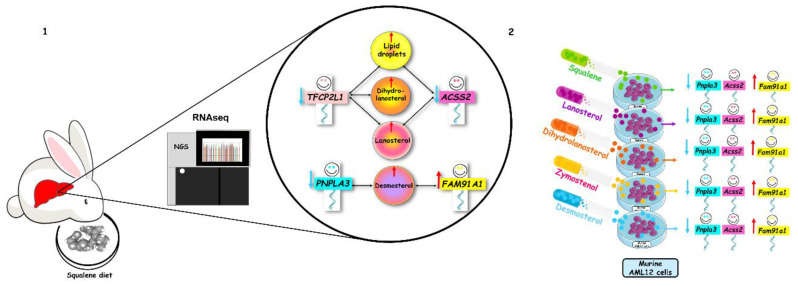
Overview of experimental approaches and main findings. (**1**) In vivo dietary intervention in rabbits and (**2**) cell culture incubation using AML12 cell line. NGS, next generation sequencing.

The nutrigenomic approach was carried out through transcriptomic analysis of the liver in male wild-type white rabbits (*Oryctolagus cuniculus*) following 0.5% squalene intake (0.6 g/kg). The administration induced increased hepatic squalene accumulation and elevated levels of post-squalene metabolites of cholesterol biosynthesis, lanosterol, dihydrolanosterol, lathosterol, zymostenol and desmosterol. Using RNA sequencing, squalene intake did not modify single nucleotide polymorphisms; however, the terpene induced alternative splicing patterns, both at 5′ and 3′ sites and retained introns. Rabbits receiving squalene displayed significant hepatic expression changes of *LOC100344884* (*PNPLA3*), *GCK*, *TFCP2L1*, *ASCL1*, *ACSS2*, *OST4*, *FAM91A1*, *MYH6*, *LRRC39*, *LOC108176846*, *GLT1D1* and *TREH*. Post-squalene metabolite levels were also associated with transcript levels. Moreover, the modified expression of *Acss2*, *Fam91a1* and *Pnpla3* was modified in the murine AML12 hepatic cell line by incubation with squalene and post-squalene intermediates in cholesterol biosynthesis such as lanosterol, dihydrolanosterol, zymostenol and desmosterol. A global overview of all experiments and results can be obtained from Figure 9. These findings indicate that transcriptional changes are not only dependent on squalene but also its downstream metabolites.

The absence of changes in body weight in animals receiving dietary squalene compared to control rabbits and the normal histomorphometric evaluation of hepatocytes (Figure 2) implies the nontoxic accumulation of the triterpene in small lipid droplets, unlike mice, where it accumulates in larger droplets [20,21]. Interestingly, a similar pattern of droplets was observed with oleanic acid triterpene [22]. These droplets could accumulate lipids in the form of non-esterified cholesterol [21], acting as energy storage depots and barriers against lipotoxicity, preventing cellular membrane defects, mitochondrial dysfunction, and errors in signaling pathways [23]. In contrast to previous studies in mice, squalene administration in rabbits did not decrease hepatic triglyceride content. Nor were there observed changes in *CYP2B* and *CYP2C* expressions (data not shown) using a similar dose to mice [9]. The length of treatment, animal model and experimental designs are crucial differences that could be responsible for the discrepant results.

Squalene mainly modified the hepatic expression for gene clusters involved in the hepatic transport of lipids and proteins, lipid metabolism, lipogenesis, protective effects against inflammation and neoplasm. These findings might contribute to explaining the absence of inflammation even in the presence of increased content of non-esterified cholesterol and increased caspase1 levels [21].

*FAM91A1*, a poorly understood gene, seems to play a regulatory role in golgin-mediated vesicle capturing in order to process, package and transport proteins and lipid molecules [24,25]. *FAM91A1* expression was not correlated to the increased squalene content (data not shown), which might be explained by the fact that this triterpene is also distributed in further subcellular organelles, including nuclear and plasma membranes and rough endoplasmic reticulum [20]. The reduced expression of *ACSS2* and *TFCP2L1* was associated with elevated lipid droplet areas in the liver (Figure 7B). The reduced expression of *GCK*, *ACSS2* and *LOC100344884* and the increased expression of *TREH* are of interest regarding the squalene-modified hepatic lipid profile. *GCK* activates lipogenesis, increases cellular triglyceride content, and regulates glucose disposal [26,27]. Reduced-*GCK* activity lowers mRNA levels for triglyceride synthesis enzymes but also for insulin receptors, leading to insulin resistance [27,28,29], suggesting that GCK inhibition by squalene may be useful in the treatment against fatty liver. *ACSS2* is a metabolic gene that promotes fat storage. *ACSS2* deficiency inhibits activity of lipid transporters and fatty acid oxidation genes, which then lowers dietary lipid absorption, reduces triglyceride content [29], and lessens hepatic fibrosis [30]. *LOC100344884*, the ortholog for *Pnpla3*, expresses lipase activity towards triglycerides in hepatocytes and retinyl esters in hepatic stellate cells [31] and was strongly associated with hepatic cholesterol content (Figure 7), fibrosis and steatosis [31,32]. *TREH* is the least studied membrane-bound α-glucosidase that hydrolyzes trehalose. Its transcript is normally expressed in intestine [33,34] and to a lesser amount in liver (https://gtexportal.org/home/gene/TREH, accessed on 1 March 2022). Squalene increased expression of *TREH* that could drive glucose metabolism [35]. Regarding protein modification, squalene-upregulated *OST4* may play a key role in promoting co-translational N-glycosylation by stabilizing STT3A-containing *OST* isoforms present in high amounts in liver cells [36]. Glycosylation deficiency interferes with protein functions and drug disposition [37,38] and reduces low-density lipoprotein cholesterol [39]. Two transcripts, *MYH6* and *LRRC39*, mainly expressed in muscle, were upregulated in the liver in response to squalene intake. Hepatocytes showed very low expression (1.6 transcripts per million vs. a median of 3.3) [40]. Interestingly, both transcript levels were associated (Figure 6) and share a function in cell signaling by protein dephosphorylation. Overexpression of (*Myh6/Ghrl*) in transgenic rats lessened oxidative stress and prevented fat dietary–induced hyperglycemia [41]. Squalene might modulate lipid and glucose metabolism through the expression of these genes.

Rabbits receiving squalene increased the hepatic content of cholesterol precursors including lanosterol, dihydrolanosterol, lathosterol and zymostenol via the Kandutsch–Russell path and desmosterol via the Bloch path. In addition, strong correlations linked squalene-modified transcript levels with increased hepatic quantities of cholesterol precursors. In this sense, the reduced expression of *TFCP2L1* was strongly associated with the accumulation of lanosterol and dihydrolanosterol, as did *ACSS2* in regard to lanosterol. The increased expression of *FAM91A1* and the decreased expression of *LOC100344884* were associated with desmosterol. These associations indicate that the observed actions of squalene could be executed through its post-squalene precursors in the cholesterol biosynthesis pathway and may differ from those observed when squalene is not metabolized [42]. Squalene accumulation in cholesterol auxotrophic lymphomas prevents oxidative cell death [42]. To solve this dilemma, the AML12 hepatic cell line was incubated with lanosterol, dihydrolanosterol, zymostenol or desmosterol, followed by analyses of *Acss2*, *Fam91a1* and *Pnpla3* transcripts. Obtained results (Figure 8) displayed the same expression changes that those observed in vivo by squalene administration. In consequence, a direct hepatic transcriptional effect of lanosterol, dihydrolanosterol, zymostenol and desmosterol is involved.

Previous studies have demonstrated that squalene shows protective anti-tumor activities [43]. ACSS2 captures acetate as a carbon source for the proliferation of hepatocellular carcinoma [28,29]. Adult mice lacking ACSS2 exhibit reduced hepatic tumor burdens [29]. TFCP2L1, a transcription factor required in germ cell specification and cholangiocyte-to-hepatocyte differentiation [44], is strongly correlated with several transcripts modulated by squalene, including OST4, TREH, GCK, ACSS2, MYH6 and LRRC39 (Figure 6). TFCP2L1 is down regulated in renal and thyroid cancer cells [45]. Besides, GLT1D1 is a tumor suppressor gene and is highly expressed in hepatocellular carcinoma [46].

In conclusion, dietary squalene accumulates in the liver and functions as a modulator of the hepatic transcriptome in wild-type *Oryctolagus cuniculus*. Concomitantly, increased levels of post-squalene metabolites of cholesterol biosynthesis, including lanosterol, dihydrolanosterol, lathosterol, zymostenol and desmosterol, were noted. Significant hepatic expression changes of *LOC100344884* (*PNPLA3*), *GCK*, *TFCP2L1*, *ASCL1*, *ACSS2*, *OST4*, *FAM91A1*, *MYH6*, *LRRC39*, *LOC108176846*, *GLT1D1* and *TREH* were observed in rabbits receiving squalene. Many of these changes were correlated with post-squalene metabolite levels. Furthermore, incubation of the murine AML12 hepatic cell line in the presence of lanosterol, dihydrolanosterol, zymostenol and desmosterol modified *Acss2*, *Fam91a1* and *Pnpla3* expressions. These findings indicate that squalene and post-squalene metabolites play important roles in hepatic transcriptional changes.

## 4. Materials and Methods

### 4.1. Animal Models and Experimental Design

The experimental animals used were 10 male wild-type New Zealand white rabbits obtained from *Servicio de Apoyo a la Investigación Animal* (*Universidad de Zaragoza*). Animals were divided into two groups, the first fed a control diet enriched with 1% of sunflower oil (*n* = 5) and the second fed a diet containing 1% of sunflower oil and 0.5% of squalene (Sigma-Merck, Darmstadt, Germany) (*n* = 5). Initial body weight for control and experimental groups was 1560 ± 201 and 1410 ± 203 g, respectively. Taking into account consumed food and body weight, the dose was equivalent to 0.6 mg/kg/day [20]. Fresh diets were prepared weekly and changed each 2 days to reduce oxidation of squalene. The animals were fed the experimental diets for 4 weeks and they were well tolerated.

Body mass and dietary intake were recorded every 2 days. After dietary intervention, rabbits were starved for 18 h, then weighed and euthanized by cervical dislocation, and livers were obtained. An aliquot of each sample was stored in neutral formaldehyde and the remaining was frozen immediately in liquid nitrogen.

### 4.2. Liver Histology Analyses

The liver samples stored in formaldehyde were then embedded in paraffin wax 4 μm sections were stained with hematoxylin and eosin and observed under Nikon microscope. Lipid droplets were estimated by quantifying as percentage of total liver section with Adobe Photoshop CS3 (Adobe Inc., San Jose, CA, USA).

### 4.3. Quantification of Hepatic Lipids

10 mg sections of liver were used to extract and quantify lipids as previously mentioned [21].

### 4.4. Quantification of Hepatic Sterols

Sterol extraction and quantification by as chromatography and mass spectrometry was performed as previously described [47].

### 4.5. Quantification of Hepatic Squalene

Squalene was isolated and quantified by solid phase extraction, gas chromatography and mass spectrometry (GC/MS) as mentioned [20].

### 4.6. RNA Extraction

Total liver RNA was extracted using Quick-RNA MiniPrep kit (Cat. No: R1055, ZYMO Research, Irvine, CA, USA) following manufacturer’s instructions. Extracted RNA was quantified by absorbance at A_260_/_280_ with Nanodrop Spectrophotometer. The purity was determined by analysis of absorbance at A260/A280 the ratio was ~2. The integrity of both 28S and 18S ribosomal RNAs was verified by agarose gel electrophoresis followed by ethidium bromide staining and the 28S/18S ratio was higher than 2.

### 4.7. RNAseq and Data Analyses

#### 4.7.1. Library Construction and Sequencing Was Carried Out by BGI (Shenzhen, China) Service

Briefly, poly-A containing mRNA molecules were purified using poly-T oligo-attached magnetic beads. Following purification, mRNA was fragmented into small pieces using divalent cations under elevated temperatures. Cleaved RNA fragments were copied into first-strand cDNA using reverse transcriptase and random primers. This was followed by second-strand cDNA synthesis using DNA polymerase I and RNase H. These cDNA fragments then have the addition of a single ‘A’ base and subsequent ligation of the adapter. The products were then purified and enriched with PCR amplification. Then PCR yield was quantified using Qubit samples then pooled together to make a single-strand DNA circle (ssDNA circle), which gave the final library. DNA nanoballs (DNBs) were generated with the ssDNA circle by rolling circle replication (RCR) to enlarge the fluorescent signals at the sequencing process. The DNBs were loaded into the patterned nanoarrays, and pair-end reads of 100 bp were read through on the DNBseq platform for the following data analysis study. For this step, the DNBseq platform combines the DNA nanoball-based nanoarrays and stepwise sequencing using Combinational Probe-Anchor Synthesis Sequencing Method.

#### 4.7.2. Bioinformatics Workflow

Sequencing reads containing low-quality, adaptor-polluted and high amounts of unknown base reads were removed, then these reads were mapped onto reference genome (*Oryctolagus_cuniculus* GCA_000003625.1) using HISAT2 followed with novel gene prediction using StringTie to reconstruct transcripts, and Cuffcompare to compare reconstructed transcripts to reference annotation. Moreover, CPC was used to predict coding potential of novel transcripts, followed by merging coding novel transcripts with reference transcripts to obtain a complete reference. With genome mapping results, GATK was used to call SNP and INDEL for each sample, then filter out the unreliable sites results in final SNP and INDEL report. RMATS was also used to detect differentially splicing genes (DSG) between samples. After novel transcript detection, novel coding transcripts were merged with reference transcripts to obtain complete reference, then clean reads were mapped to reference using Bowtie and gene expression level for each sample was calculated with RSEM. This was followed by clustering analysis and functional annotations. The complete database was deposited in the GEO database (GSE191236).

### 4.8. Quantification of mRNA

RNAseq transcripts displaying signal log_2_ ratio > 1.5 or <−1.5 and false discovery rate < 0.001 were selected for confirmation and 18 out of 22 genes fulfilling these criteria assayed by reverse transcriptase quantitative PCR (RT-qPCR) assay. Primers for specific sequences were designed using NCBI and then were checked for gene specificity and full variant coverage by BLAST (NCBI), KEGG and Ensemble Genome Browser. Equal amounts of (500 ng) DNA-free RNA were used to synthesize complementary DNA using PrimeScript RT reagent Kit (Cat. No: RR037A, Takara, Kutsatsu, Shiga, Japan) following manufacturer’s instructions. Primers’ concentrations and cDNA input were optimized to obtain efficiencies in the range of 90% to 110% (Supplementary Table S1). Quantitative real-time was carried out using SYBR Green dye master mix (Applied Biosystems, Foster City, CA, USA) according to manufacturer’s instructions, utilizing Step One Real-Time PCR System (Applied Biosystems, Foster City, CA, USA). Analysis of relative gene expression data was calculated using the 2^(−ΔΔCT)^ method and normalized to the average of both reference genes *PPIB* and *GAPDH* for rabbits. *Ppib* was used as a reference gene for the murine AML12 cell line.

### 4.9. AML12 Cell Culture

Hepatic cell line of murine origin was grown in a humidified atmosphere of 5% CO_2_ at 37 °C in Dulbecco’s modified Eagle’s minimum essential medium (DMEM) (Thermo Fisher Scientific, Waltham, MA, USA): F12-Ham’s medium (GE Healthcare Life Science, South Logan, UT, USA) enriched with fetal bovine serum and insulin/transferrin/selenium. When cells reached a confluence of 90–100%, medium was removed, cells were washed twice with phosphate buffered saline followed by addition of medium free of fetal bovine serum, insulin, transferrin and selenium. Cells were then incubated for 6 h with 200 µM squalene (Sigma-Merck, Darmstadt, Germany) or with 200 nM lanosterol, dihydrolanosterol, zymostenol or desmosterol (Avanti Polar lipids, Alabaster, AL, USA). Each condition was tested in six replicates. Media were removed, cells were washed twice with phosphate buffered saline then collected and total RNA was extracted using Tri-reagent solution (Ambion, Austin, TX, USA). DNA contaminants were removed by TURBO DNAse treatment using DNA removal kit (Ambion, Austin, TX, USA). Squalene and sterol effects were investigated at mRNA level by RT-qPCR assays.

### 4.10. Quality Control and Statistics

Samples in quantitative real time were tested in duplicate and their coefficient of variation obtained. Duplicates showing coefficient of variation higher than 3% were discarded and tested again. Statistical analyses were performed with GraphPad Prism (GraphPad Software, San Diego, CA, USA). Data was analyzed for normal distribution by Shapiro-Wilk’s test and homogeneity of variance by Bartlett’s F-test. When any of these parameters failed, results were analyzed by Mann–Whitney’s U test. Differences in both groups were considered significant if *p* < 0.05. Correlation among tested results was analyzed using Spearman’s ρ correlation coefficient.

## Figures and Tables

**Table 1:** Hepatic transcripts differentially downregulated by the administration of squalene at the level of signal log_2_ ratio < 1.5 and false discovery rate < 0.001 in male *Oryctolagus cuniculus* according to RNAseq.

Biological Process	GenBank	Name	Gene Symbol	Signal log_2_ Ratio	*p*-Value
Intracellular protein transport	XM_017339423.1	B-cell receptor-associated protein 29, BCAP29	*LOC100344375*	−2.8	0.00000
Hydrolysis of triglycerides	XM_017339724.1 XM_008252198.2 XM_008252200.2 XM_017339725.1 XM_008252201.2	Patatin-like phospholipase domain-containing protein 3, PNPLA3	*LOC100344884*(*PNPLA3*)	−1.9	0.00018
Glucose metabolism	XM_008261818.2	Glucokinase	*GCK*	−1.7	0.00061
LncRNA	XR_001795369.1 XR_001795370.1 XR_519422.2	Uncharacterized LOC103351691	*LOC103351691*	−1.6	0.00026
Regulation of transcription	XM_008251077.2	Transcription factor CP2 like 1	*TFCP2L1*	−1.6	0.00001
Fatty acid biosynthesis	XM_017339196.1	Acetyl-CoA carboxylase beta	*ACAB*	−1.6	0.00004
Transcription activity	XM_002711229.3	Achaete-scute family bHLH transcription factor 1	*ASCL1*	−1.5	0.00246
NA	XM_017348395.1	E3 ubiquitin-protein ligase HERC2-like	*LOC108178363*	−1.5	0.00225
Acetate-CoA ligase activity	XM_002710791.3 XM_002710792.3	Acyl-CoA synthetase short chain family member 2	*ACSS2*	−1.5	0.00273

**Table 2:** Hepatic transcripts differentially upregulated by the administration of squalene at the level of signal log_2_ ratio > 1.5 and false discovery rate < 0.001 in male *Oryctolagus cuniculus* according to RNAseq.

Biological Process	GenBank	Name	Gene Symbol	Signal log_2_ Ratio	*p*-Value
Protein glycosylation	XM_017346007.1	Dolichyl-diphosphooligosaccharide―protein glycosyltransferase subunit 4, OST4	*LOC108177690* (*OST4*)	2.5	2.60 × 10^−7^
Intracellular protein transport, vesicle tethering to Golgi	XM_002710763.3 XM_008255883.2 XM_008255884.2 XM_017341515.1	Family with sequence similarity 91 member A1	*FAM91A1*	2.3	4.39 × 10^−6^
NA	XR_515397.2	Uncharacterized LOC103345531	*LOC103345531*	2	0.00009
Actin binding, ATP binding	XM_017348206.1	Myosin-6	*MYH6*	1.8	0.00041
Regulate bone mineralization	NM_001101695.1	Osteomodulin	*OMD*	1.8	0.00013
Negative Regulation of translational initiation	NM_001204114.1	Eukaryotic translation initiation factor 4E binding protein 3	*EIF4EBP3*	1.7	0.00046
Integral component of membrane	XM_008249188.2 XM_017337996.1 XM_008249187.2 XM_008249192.2 XM_008249193.2 XM_008249194.2	Leucine rich repeat and Ig domain containing 1	*LINGO1*	1.7	0.00022
NA	XM_002715469.3	Leucine rich repeat containing 39	*LRRC39*	1.6	0.00086
Nucleoside triphosphate catabolic process, immune response	XM_008263526.2 XM_017345377.1	Ectonucleotide pyrophosphatase/phosphodiesterase 3	*ENPP3*	1.6	0.00022
lncRNA	XR_001793580.1 XR_001793581.1	Uncharacterized LOC108176846	*LOC108176846*	1.6	0.00009
Muscle contraction	XM_017343215.1	Titin	*TTN*	1.6	0.00133
Glycosylation	XM_008251202.1	Glycosyltransferase 1 domain containing 1	*GLT1D1*	1.5	0.00002
Trehalose metabolism	NM_001082290.1	Trehalase	*TREH*	1.5	0.00057

**Table 3:** Changes in selected hepatic gene expressions of male *Oryctolagus cuniculus* receiving 0.5 mg/kg squalene/day according to RT-qPCR assay for genes with signal log_2_ ratio < −1.5 or >1.5.

Gene Symbol	Control (*n* = 5)	Squalene (*n* = 5)	Fold Change	Signal log_2_ Ratio
*ASCL1*	2.4 ± 3.4	0.1 ± 0.2 *	0.1	−4.2
*ACAB*	2.3 ± 3.3	0.5 ± 0.3	0.2	−2.3
*GCK*	1.5 ± 1.4	0.3 ± 0.2 *	0.2	−2.2
*LOC100344884* (*PNPLA3*)	1.2 ± 0.6	0.3 ± 0.3 *	0.3	−1.9
*ACSS2*	1.0 ± 0.2	0.3 ± 0.2 **	0.3	−1.8
*TFCP2L1*	1.0 ± 0.2	0.4 ± 0.2 **	0.4	−1.3
*LOC100344375*	1.5 ± 1.1	0.9 ± 0.5	0.6	−0.7
*LOC103351691*	2.0 ± 2.1	1.9 ± 1.7	1.0	0.0
*FAM91A1*	1.1 ± 0.6	2.0 ± 0.9 *	1.8	0.8
*LOC108177690* (*OST4*)	1.1 ± 0.5	2.2 ± 0.8 *	2.0	1.0
*LRRC39*	1.1 ± 0.4	2.0 ± 0.9 *	1.9	1.0
*LOC108176846*	1.4 ± 1.2	3.6 ± 1.9 *	2.6	1.4
*GLT1D1*	1.4 ± 1.2	4.4 ± 2.3 *	3.2	1.7
*MYH6*	2.2 ± 1.9	11.2 ± 9.7 *	5.1	2.3
*OMD*	4.1 ± 5.6	19.7 ± 28.2	4.8	2.3
*TTN*	1.0 ± 2.1	5.8 ± 5.3	5.6	2.6
*TREH*	1.4 ± 1.1	16.3 ± 15.2 *	11.7	3.6

Results are expressed as means and standard deviations normalized to *PPIB* and *GAPDH*. Statistical analyses were carried out according to Mann–Whitney U-test and *, *p* < 0.05, ** *p* < 0.01 vs. control.

## Data Availability

Data is contained within the article and supplementary material and RNA seq dataset is deposited at GEO (GSE191236).

## Supplementary Materials

The following supporting information can be downloaded at: https://www.mdpi.com/article/10.3390/ijms23084172/s1.
