# Supplementary material for: Squalene through Its Post-Squalene Metabolites Is a Modulator of Hepatic Transcriptome in Rabbits
Source: Int J Mol Sci. 2022 Apr 10;23(8):4172. doi: 10.3390/ijms23084172 (PMC9031321; doi:10.3390/ijms23084172)
Supplement: Supplementary file 1 [file ijms-23-04172-s001.zip › ijms-1670349-Supplementary.pdf]

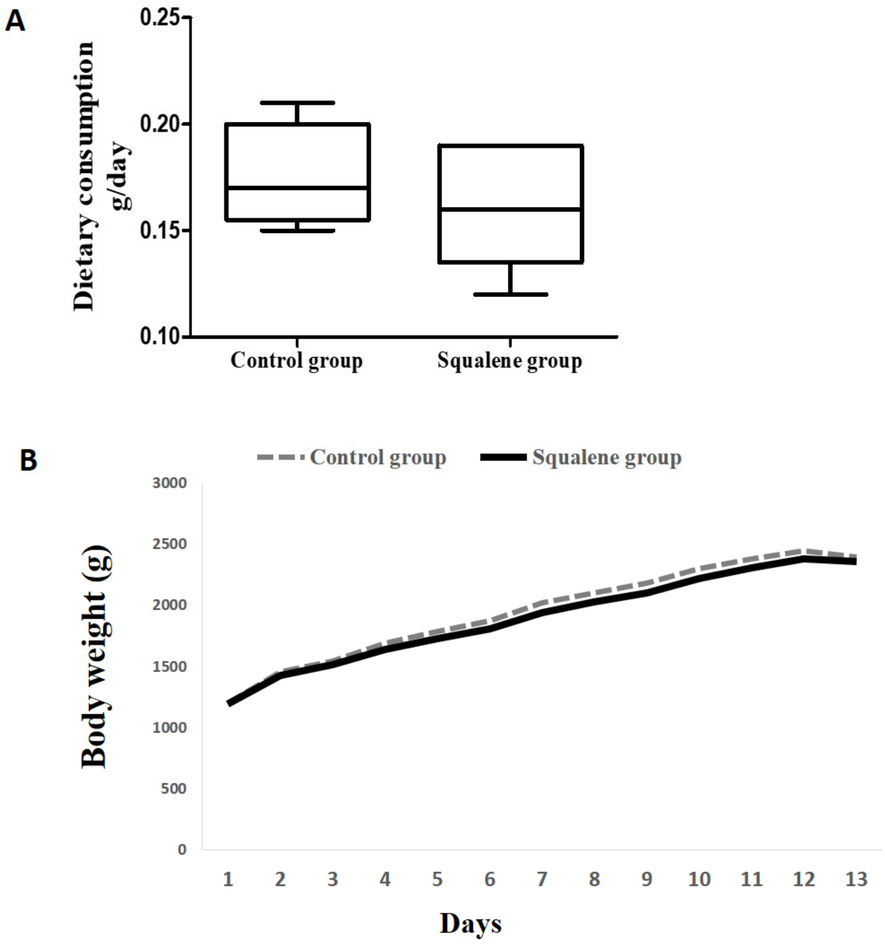

Supplementary Figure S1. Body weight difference in response to dietary consumption. (**A**) Food consumption of both groups. Statistical analyses was done according to Mann-Whitney's U-test. $p > 0.05$. (**B**) Body weight for control and squalene groups was compared among different days of assay.

Supplementary Table S1. Characteristics of primers used in RT-qPCR according to MIQE guidelines.

Gene symbol	Accession		Sequence	Amplicon length	Exons	[Primer]	Efficiency
**Oryctolagus cuniculus**							
LOC100344375	XM_017339423.1	Sense	GTTGAGAAGGGCTCAACCGT	126	4/5	0.3 µM	97%
		Antisense	CAGACGTCTCAACACAAGCC				
LOC100344884	XM_017339724.1	Sense	CCTTCTACGGGGAGCATGAC	169	5	0.3 µM	96%
		Antisense	TACCCGCGAACGCATATCTC				
GCK	XM_008261818.2	Sense	AGACATCGACAAGGGCATCC	79	4/5	0.3 µM	90%
		Antisense	CCCCACGATGTTGTTCCCTT				
LOC103351691	XR_001795369.1	Sense	AACCAGCCCAGAAGATGACTG	136	NA	0.3 µM	100%
		Antisense	CCTGTGGTCTTCCTTTGCTCT				
TFCP2L1	XM_008251077.2	Sense	CGAAGAGACGCTGACCTACC	212	2/4	0.3 µM	93%
		Antisense	CAATGTCCAGGATGCGGTCT				
ACACB	XM_017339196.1	Sense	GGCCATCCGGTTTGTAGTCA	125	3/4	0.3 µM	99%
		Antisense	CCACGTTGGCGTAGTTGTTG				
ASCL1	XM_002711229.3	Sense	TTGGTGCGAATGGACTTTGG	70	1/2	0.3 µM	94%
		Antisense	CGTCACTGACCAGAAAGCAC				
ACSS2	XM_002710791.3	Sense	AACAGGGCATTCGAAAGGGT	127	4/5	0.3 µM	98%
		Antisense	GCAGAGAAGCCTGCAAACAC				
LOC108177690	XM_017346007.1	Sense	CTGTGTGGAGGCAGTGTAGG	113	1	0.3 µM	98%
		Antisense	GAGAACGACGAGCAGGAAGA				
FAM91A1	XM_002710763.3	Sense	CAAGGTGGACTTGCAGCATT	236	20/22	0.3 µM	99%
		Antisense	CAAGAGGAACCCAGTCTGCC				
MYH6	XM_017348206.1	Sense	GACACTGGCAAAGGCAAAGG	234	NA	0.3 µM	104%
		Antisense	CTTCCTGCAGATGCGAATGC				
OMD	NM_001101695.1	Sense	TCAACCCCTGAAAACTAACGC	157	1/2	0.3 µM	103%
		Antisense	CCTCACTTGGGTCTTGGTCAT				
LRRC39	XM_002715469.3	Sense	ACGAGGGAGGATGGAAGAGT	219	3	0.4 µM	96%
		Antisense	AGTGAGCAGTCCAATTCCTCG				
LOC108176846	XR_001793580.1	Sense	TCTGGGGACCTTACGGAGTC	107	NA	0.3 µM	93%
		Antisense	TCAGCTCCTGAGAAAGTGCG				
TTN	XM_017343215.1	Sense	TGCCAAGTGACCAACCTTGT	186	271/272	0.3 µM	93%
		Antisense	GGCTAAGGTCGCACTGTTCT				
GLT1D1	XM_008251202.1	Sense	AGAGTCAAGGCATCACCACG	89	2/4	0.3 µM	91%
		Antisense	GTCGTCAGCACTCCGGTTTA				

TREH	NM_001082290.1	Sense	CACCCTGTGAGAGCCAGATT	81	1/2	0.3 µM	99%
		Antisense	GCTTATCGTCCGGGTAGAGC				
GAPDH	NM_001082253.1	Sense	TCGGAGTGAACGGATTTGGC	146	NA	0.3 µM	105%
		Antisense	GCCGTGGGTGGAATCATACT				
PPIB	XM_002718143.4	*Sense*	TCCCAGTTCTTCATCACCACAG	83	4	0.3 µM	94%
		Antisense	TCCATGCCCTCCAGAAACTTT				
**Gene symbol**	**Accession**		**Sequence**	**Amplicon length**	**Exon**	**[Primer]**	**Efficiency**
**Mice AML12 cell line**							
Fam91a1	NM_145959.3	Sense	TGGCGCCTCTTACCAATGAG	124	16/17	0.1 µM	99%
		Antisense	CCTTGCCCAGTGACATGGTA				
Acss2	NM_019811.3	Sense	GCCATATGCTGACCCCTCTC	103	12/13	0.3 µM	100%
		Antisense	TCCCCGGACTCATTCAGGAT				
Pnpla3	NM_054088.3	Sense	CTTCCTGGGCTTCTACCACG	152	1/2	0.3 µM	103%
		Antisense	CCATTATACGGCCGAGAGGG				
Pipb	NM_011149.2	Sense	GGAGATGGCACAGGAGGAA	72	3/4	0.1 µM	103%
		Antisense	GTAGTGCTTCAGCTTGAAGTTCTCAT				
